# Association of Human Leukocyte Antigen Polymorphisms With Moderate-to-Severe Dry Eye With and Without Sjögren's Syndrome

**DOI:** 10.1167/iovs.66.9.57

**Published:** 2025-07-22

**Authors:** Zhiqing Chen, Dan Jiang, Qinxiang Zheng, Shilai Xing, Jinyang Li, Lei Lin, Li Ren, Hanlu Xu, Xiaoguang Yu, Louis Tong, Wei Chen

**Affiliations:** 1 National Clinical Research Center for Ocular Diseases, Eye Hospital, Wenzhou Medical University, Wenzhou, China; 2 Institute of PSI Genomics, Wenzhou Global Eye & Vision Innovation Center, Wenzhou, China; 3 Cornea and External Eye Disease Service, Singapore National Eye Center, Singapore; Ocular Surface Research Group, Singapore Eye Research Institute, Singapore; Eye-Academic Clinical Program, Duke - National University of Singapore, Singapore, Singapore

**Keywords:** dry eye, Sjögren's syndrome (SS), human leukocyte antigen (HLA), genetic susceptibility, clinical manifestations

## Abstract

**Purpose:**

Sjögren’s syndrome dry eye (SSDE) has been described in relation to human leukocyte antigen (HLA) polymorphisms. It is unknown whether non-Sjögren’s syndrome dry eye (NSDE) is related to HLA. This study aimed to evaluate the genetic association of HLA polymorphism with dry eye disease (DED). This was a cross-sectional study.

**Methods:**

Patients with moderate-to-severe DED with and without Sjögren’s syndrome (SS) were included. Oral swab samples were collected and HLA genotyping was performed using single-molecule real-time sequencing. The normal healthy population was used as the control group for the susceptibility alleles identification. The signs and symptoms of DED were compared between patients with and without susceptibility alleles.

**Results:**

Sixty-four (64) patients with NSDE (mean age = 47.55 years, 92.2% female) and 40 patients with SSDE (mean age = 52.68 years, 90% female) were included. HLA-B*40:01 allele frequency in the NSDE group was significantly higher than in the reference population (odds ratio [OR] = 2.42, *P* < 0.001). We identified a trend toward worsening of the ocular surface condition with HLA-B*40:01 carrier. This was manifested by a higher Oxford score (*P* < 0.0001) and more meibomian gland dropout (*P* = 0.030) and worse visual acuity (*P* = 0.006). Further analysis revealed that higher severe rate of NSDE was associated with HLA-B*40:01 carrier status (67.39% vs. 42.68%, *P* = 0.007). In addition, patients with SSDE showed a significantly higher frequency of HLA-B*40:01, HLA-C*07:02, HLA-DQB1*06:01, and HLA-DRB1*16:02. Patients with SSDE carrying HLA-B*40:01 had less tear secretion.

**Conclusions:**

Significant associations exist between HLA alleles and moderate-to-severe DED. HLA polymorphisms were associated with the clinical features of moderate-to-severe DED, possibly via modulating the ocular surface immune function.

Dry eye disease (DED) is a multifactorial ocular surface disease that causes eye discomfort and visual impairment.^1^ According to the Tear Film and Ocular Surface Society Dry Eye Workshop (DEWS) epidemiology subcommittee, the prevalence of DED varies widely across studies, from 5% to as high as 50%.^2^ Their consensus was that the prevalence of moderate-to-severe DED is approximately 5%.^3^ Moderate-to-severe DED involves serious ocular surface damage characterized by a lingering and recurrent course and a poor prognosis. It can lead to visual dysfunction in severe cases, and can seriously affect the quality of life of patients and become an important public health concern.

Autoimmune disease-associated dry eye is one of the important types of DED and Sjögren’s syndrome (SS) is the most common. SS is a systemic autoimmune exocrine inflammatory disease that mainly affects the salivary and lacrimal glands, and manifests as moderate-to-severe dry eye and xerostomia.^4^ The prevalence of SS worldwide ranges from 0.05% to 0.6%.^5^ SS is considered a multifactorial disease with complex interactions between the genetic background and environmental factors.^6^ Many studies have described an association of human leukocyte antigen (HLA) polymorphisms with SS.^7^ HLA, the major histocompatibility complex (MHC) in human beings, is closely related to regulation of the immune response. It exhibits the highest degree of polymorphisms with thousands of single nucleotide polymorphisms (SNPs) that are distributed across various MHC loci in three subtypes, significantly influencing antigen presentation specificity by altering peptide binding motifs.^8^ Studies suggest that the MHC phenotype of the antigen presenting cell can modulate the Th1/Th2 balance and lead to resistance or susceptibility to the disease. With the increasing research in immunology and genetics at a molecular level, the close relationship between HLA and autoimmune disease has become increasingly clear.^9^^–^^11^

Clinically, partial patients with moderate-to-severe dry eye present in the absence of autoimmune disorders. DED is at least in part genetic.^12^ Indeed, immunological inflammatory responses of the ocular surface are considered one of the key mechanisms of DED, widely considered an excessive T-cell mediated inflammatory response.^13^^–^^15^ Intense conjunctival expression of the HLA-DR antigen has been demonstrated in patients with dry eye. Cyclosporine treatment does decrease HLA-DR immunoreactivity.^16^ There are indeed similarities between non-Sjögren's syndrome dry eye and Sjögren’s syndrome dry eye (NSDE and SSDE, respectively), including symptoms, signs, and medical history. Akpek EK et al. estimated the prevalence of SS could reach 10% among patients with moderate-to-severe DED.^17^^,^^18^ Almost all patients have an average 10-year history of DED before diagnosis.^19^ However, whether HLA polymorphisms are associated with NSDE is not clear.

Hence, we aimed to discover further insights about the potential association between HLA polymorphisms and moderate-to-severe DED, including NSDE and SSDE, as well as the presence of shared HLA polymorphisms between the two. Consequently, we formulated a hypothesis that HLA polymorphisms modulate susceptibility to moderate-to-severe DED. In this study, noninvasive sampling and high-resolution typing were applied to obtain the HLA allele frequencies in Chinese Han individuals afflicted with moderate-to-severe DED with and without SS. In addition, a comparative analysis was conducted to determine the distribution of HLA alleles between patients and healthy individuals, and to clarify the contribution of risk HLA alleles to disease susceptibility and clinical manifestations.

## Methods

### Study Population

This cross-sectional study included 104 consecutive patients diagnosed with moderate-to-severe dry eye at Wenzhou Medical University Eye Hospital, from May 2021 to August 2023. The study was approved by the Ethics Committee of Wenzhou Medical University Eye Hospital (approval no. 2021-059-K-51/2022-068-K-50) and adhered to the tenets of the Declaration of Helsinki. Written informed consent was obtained from all patients. They were not related and were recruited from the outpatient department. The diagnosis of DED was based on the DEWS II Diagnostic Methodology Subcommittee criteria,^20^ as defined by the Ocular Surface Disease Index (OSDI) ≥13 followed by at least 1 of homeostasis markers (non-invasive[fluorescein] tear breakup time <10 seconds or ocular surface staining >5 corneal spots or Schirmer test <10 mm/5 minutes). Moreover, at least one of the following criteria had to be met, as detailed in Table 1. Using autoantibody test results and/or labial gland biopsy findings from the tertiary hospital, patients who fulfilled the 2012 American College of Rheumatology classification criteria (see Table 1) for SS were classified as the SSDE group.^21^ In addition, those without a history of autoimmune disease and with normal blood test results (rheumatoid factor and autoimmune antibodies) were included in the NSDE group.

**Table 1.** Classification Criteria of Moderate-to-Severe DED and Sjögren’s Syndrome


Classification criteria of moderate-to-severe DED: • Ocular surface disease index (OSDI) ≥23, with one or more of the following dry eye symptoms often or all of the time: dryness, itching, burning, photophobia, pressure, gritty sensation, and blurred vision • Abnormal noninvasive tear breakup time (NIBUT) ≤5 s • Abnormal Schirmer test without local anesthesia ≤5 mm/5 min • Positive corneal staining (severe punctate erosions, confluent spots, or with filaments, Oxford staining grade ≥2)
2012 American College of Rheumatology classification criteria for Sjögren’s syndrome (at least 2 out of 3 of the following objective tests): • Positive serum anti-SSA (Ro) and/or anti-SSB (La) or positive RF and ANA ≥ 1:320 • Ocular staining score ≥ 3 • Labial salivary gland with focal lymphocytic sialadenitis and focus score of ≥1 foci/4 mm^2^

Patients were excluded if they had a history of other autoimmune disease; use of contact lenses in the previous 4 weeks; and eye surgery within 3 months; punctal plug implantation within 6 months; active ocular infection or inflammation; abnormal eyelid function and severe blepharitis.

### Questionnaires and Tests to Evaluate Dry Eye

Patient demographic information and clinical history were documented. First, the OSDI questionnaire survey and visual acuity (VA) were assessed to quantify dry eye subjective symptoms. Next, standardized dry eye tests in the following order were performed: tear meniscus height (TMH), bulbar redness, noninvasive tear break-up time (NIBUT), score of corneal fluorescence staining (CFS) according to Oxford scheme,^22^ Schirmer test without local anesthesia (approximately 30 minutes after corneal staining), and infrared imaging of the meibomian gland to obtain the meibomian gland dropout score (MGDS) based on a four-grade scoring system (0–3).^23^

### Sample Collection

Patients were instructed to refrain from food and drink consumption and smoking 30 minutes prior to collection of oral swab samples. Samples were obtained from the left and right inner cheek of each patient by swabbing with a sterile sponge (LAKEbio, Lakebio Biotechnology Co., Ltd., Hefei, China) at least 10 times. Each swab was placed in a test tube containing sample-preservation fluid (LAKEbio, Lakebio Biotechnology Co., Ltd., Hefei, China) and sealed. After collecting the sample, it can be stored and transported at room temperature.

### HLA Genotyping

DNA samples were extracted from oral swab samples stored in a cell preservation tube (LAKEbio Co., Ltd., Cat. 3bS0107.0-01.0-50) using a commercial extraction kit (Magen Biotech Co., Ltd., Cat. IVD3102-BD) and stored at −80°C. Targeted gene sequences were amplified from DNA samples by using long-fragment PCR amplification reagents and specific primers for HLA-A, HLA-B, HLA-C, HLA-DQA1, HLA-DQB1, HLA-DPA1, HLA-DPB1, HLA-DRB1, and HLA-DRB3-5 regions. At least 200 ng PCR products were input for library construction using the PacBio dedicated library construction reagents. Libraries were further mixed, and finally sequenced on the Sequel II sequencer (PacBio SMRT) based on the Circular Consensus Sequencing Mode (CCS)^24^^–^^26^ at a concentration of 100 pM.

High fidelity (HiFi) sequencing was performed to obtain CCS reads (HiFi reads). Split HiFi data (PacBio Lima software) was used for primary quality control and to filter out other species data and non-HLA data, and extract coding sequences (CDS). To ensure the accuracy of the genotyping results, it was required that the number of Q30 HiFi reads for each gene exceeds 100. These CDS sequences were compared with the IPD-IMGT/HLA database and the best ratio pairs reads were selected as candidate subtypes.^27^ The candidate subtypes were then arranged in proportion from high to low, and provided the first two subtypes and the first four subtypes (top 4); clustering software (PacBio PBAA software) was used to obtain cluster results, top 4, and cluster results for preliminary check. Haploid verification was performed on all homozygous genes in the check. Complete data analysis was performed of the corresponding HLA genes using the data obtained from sequencing, and provided blood typing results with a three-field (6-digit) high resolution allele genotyping.

### Statistical Analysis

Data for the reference population was obtained from unrelated healthy volunteer donors from the China Marrow Donor Program (CMDP).^28^ There were 812,211 individuals in the control group with 159 common and 517 well-documented alleles at the HLA-A, -B, -C, -DRB1, and -DQB1 loci with a 2-field (4-digit) allele genotyping. The frequency of Han ethnicity was 94.73% and all individuals were 18 to 45 years old. This has served as the main reference for researchers investigating HLA diversity in the Chinese population.

The HLA alleles detected in the study sample were described and compared with those in the control group. HLA allele frequencies were calculated by direct counting and determined using the formula alleles/ (2 × N). To determine differences in allele frequencies between the study sample and the reference population, we conducted Pearson’s Chi-square (χ2) test using R software (version 4.3.2). The corrected *P* value (*P*c) was obtained by multiplying the number of alleles shared by the case and control groups at each locus using the Bonferroni correction method. The association of each selected HLA allele with dry eye symptoms and signs was evaluated. Mean (SD) or median (interquartile range [IQR]) was used for continuous data, and the percentage for categorical data (i.e. gender). Between-group differences were compared using Student's *t*-test for independent samples, the Mann–Whitney test, or the χ^2^ test. Statistical analyses were performed by IBM SPSS Statistics software (version 24.0). A *P* value < 0.05 was considered statistically significant.

## Results

### Clinical Characteristics of Study Population

A total of 104 oral swabs were collected from 64 patients with NSDE and 40 patients with SSDE. The mean age of the patients with NSDE and the patients with SSDE were 47.55 ± 10.70 and 52.68 ± 12.52 years, respectively. Fifty-nine patients (92.2%) were women in the NSDE group and there were 36 (90%) women in the SSDE group. All patients were examined with the Keratograph (Oculus, Germany) to evaluate the TMH, NIBUT, bulbar redness, and MGDS. In addition, OSDI, VA, CFS, and Schirmer test were recorded. The demographic and clinical characteristics of the study population are presented in Table 2.

**Table 2.** Clinical Characteristics of Non-Sjögren's Syndrome Dry Eye Group and Sjögren’s Syndrome Dry Eye Group

Variables	Non-Sjögren's Syndrome Dry Eye (*N* = 64, 128 Eyes)	Sjögren's Syndrome Dry Eye (*N* = 40, 80 Eyes)	*P* Value
Age, mean (SD), y	47.6 (10.7)	52.7 (12.5)	0.028^*^
Female, *N* (%)	59 (92.2)	36 (90.0)	0.730^†^
OSDI score, mean (SD)	51.9 (22.3)	67.8 (20.8)	0.001^*^
VA, median (IQR), LogMAR	0.10 (0.00–0.22)	0.22 (0.05–0.43)	<0.001^‡^
Schirmer test, median (IQR), mm/5 min	4.0 (2.0–6.0)	2.0 (1.0–3.0)	<0.001^‡^
TMH, median (IQR), mm	0.12 (0.10–0.16)	0.10 (0.08–0.13)	<0.001^‡^
NIBUT, median (IQR), s	4.11 (2.92–6.45)	2.97 (0.00–4.50)	<0.001^‡^
Bulbar redness score, median (IQR)	1.1 (0.9–1.4)	1.4 (0.9–2.0)	0.055^‡^
MGDS, median (IQR)	1.0 (0.0–1.0)	1.0 (1.0–2.0)	<0.001^‡^
CFS score, median (IQR)	3.0 (1.0–4.0)	5.0 (4.0–5.0)	<0.001^‡^

CFS, corneal fluorescein staining (Oxford scheme); IQR, interquartile range; LogMAR, logarithm of the minimal angle of resolution; MGDS, meibomian gland dropout scores; NIBUT, noninvasive break up time; OSDI, ocular surface disease index; SD, standard deviation; TMH, tear meniscus height; VA, visual acuity.

* Independent sample *t-*test.

† *χ^2^* test.

‡ Mann-Whitney *U* test.

### HLA Typing Results

The full list of HLA-A, HLA-B, HLA-C, HLA-DQA1, HLA-DQB1, HLA-DPA1, HLA-DPB1, HLA-DRB1, and HLA-DRB3-5 allele in patients is shown in Supplementary Tables S1 and S2 in the Supplementary Material. Only alleles with a frequency >5% in the study patients and showing a significant difference between the study sample and the reference population are summarized in Table 3.

**Table 3.** Frequency (%) of HLA Alleles With *P* Value < 0.05 for Difference Between the Study Sample and the Reference Population

HLA-Alleles	Case Frequency (%)	Control Frequency (%)	Odds Ratio	95% CI	*P* Value	*P*c Value
Non-Sjögren’s syndrome dry eye
A*11:01	30.47	20.89	1.66	1.14–2.42	0.011	>0.05
B*15:02	7.81	3.55	2.30	1.21–4.39	0.018	>0.05
B*40:01	20.31	9.55	2.42	1.57–3.72	<0.001	0.007
C*07:02	21.88	15.19	1.56	1.03–2.38	0.047	>0.05
DQB1*05:02	12.50	7.36	1.80	1.06–3.03	0.040	>0.05
Sjögren’s syndrome dry eye
B*40:01	27.5	9.55	3.59	2.20–5.87	<0.001	<0.001
C*07:02	33.75	15.19	2.84	1.79–4.52	<0.001	0.001
DQB1*03:01	11.25	21.06	0.48	0.24–0.95	0.044	>0.05
DQB1*05:02	17.5	7.36	2.67	1.50–4.75	0.001	>0.05
DQB1*06:01	25	10.25	2.92	1.76–4.84	<0.001	0.003
DRB1*08:03	16.25	6.31	2.88	1.59–5.22	0.001	0.052
DRB1*16:02	13.75	3.06	5.06	2.68–9.56	<0.001	<0.001

Reference population: A total of 812,211 unrelated normal healthy volunteer donors from the China Marrow Donor Program; the allele frequencies were compared between patients and controls using the Chi-square/Fisher’s Exact test. A Bonferroni correction was used to corrected *P* values (*P*c) for multiple comparisons.

In the NSDE group, there were differences in the frequency of alleles, such as A*11:01 (*P* = 0.011), B*15:02 (*P* = 0.018), B*40:01 (*P* < 0.001), C*07:02 (*P* = 0.047), and DQB1*05:02 (*P* = 0.040) between the study sample and the reference population. After adjustment for multiple comparisons, differences remained only in B*40:01 (*P*c = 0.007) which was consequently identified as a susceptibility allele.

In the SSDE group, there were differences in the frequency of alleles between the study sample and the reference population, such as B*40:01 (*P* < 0.001), C*07:02 (*P* < 0.001), DQB1*03:01 (*P* = 0.044), DQB1*05:02 (*P* = 0.001), DQB1*06:01 (*P* < 0.001), DRB1*08:03 (*P* = 0.001), and DRB1*16:02 (*P* < 0.001). After adjustment for multiple comparisons, the differences remained for B*40:01 (*P*c < 0.001), C*07:02 (*P*c = 0.001), DQB1*06:01 (*P*c = 0.003), and DRB1*16:02 (*P*c < 0.001).

### Association of Susceptibility Alleles With Clinical Manifestations

**Figure 1.**
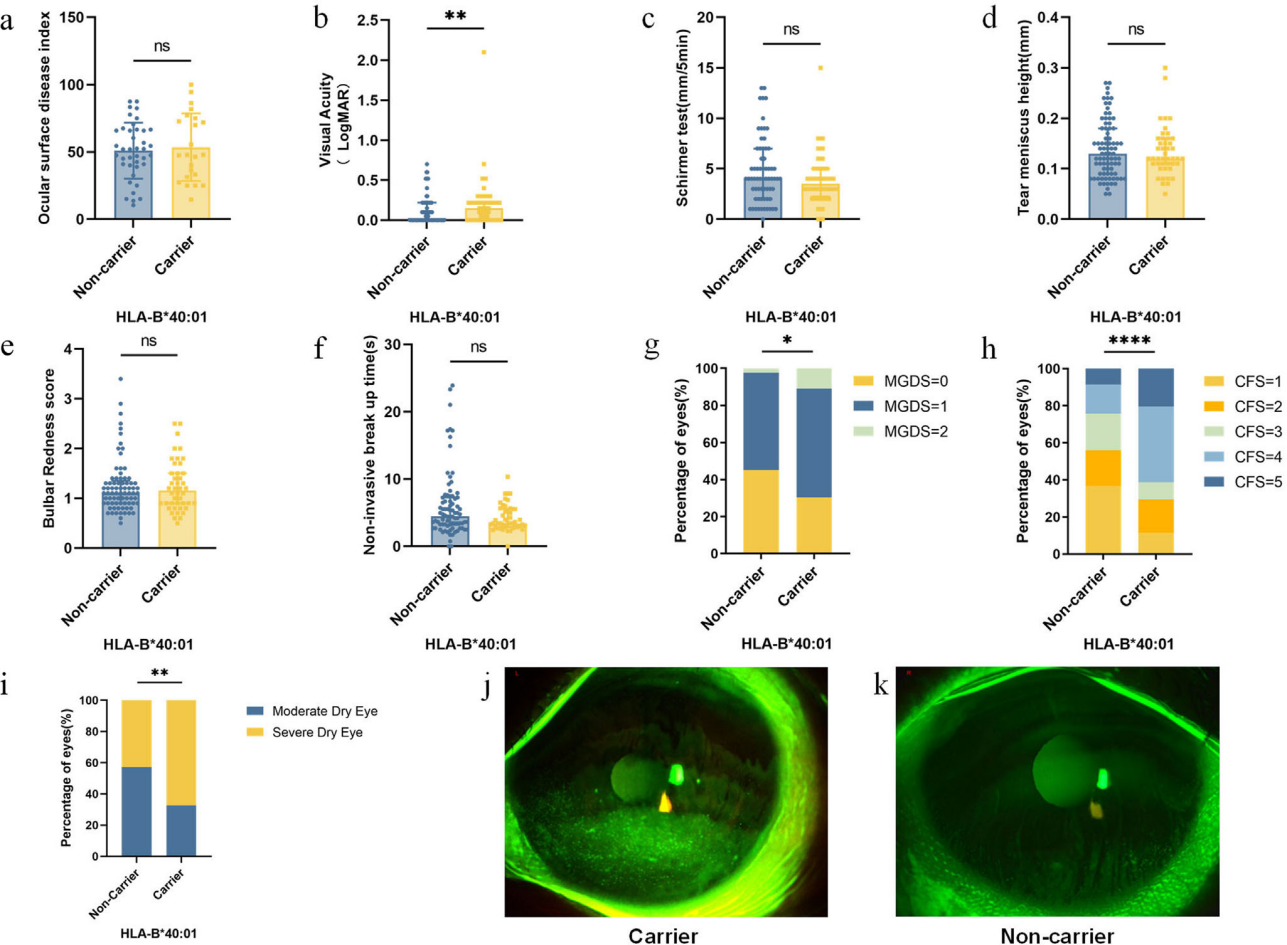
Comparison of clinical parameters between HLA-B*40:01 carrier and non-carrier patients in non-Sjögren’s syndrome dry eye group. (**a**) Ocular surface disease index (*P* = 0.655); (**b**) visual acuity (*P* = 0.006); (**c**) Schirmer test (*P* = 0.288); (**d**) tear meniscus height (*P* = 0.880); (**e**) bulbar redness (*P* = 0.901); (**f**) noninvasive break up time (*P* = 0.115); (**g**) meibomian gland dropout scores (MGDS; *P* = 0.030); (**h**) corneal fluorescein staining (CFS; *P* < 0.0001); (**i**) comparison of the higher severe rate between patients with HLA-B*40:01 carrier and non-carrier NSDE (*P* = 0.007); and (**j, k**) representative images of fluorescein staining in patients with HLA-B*40:01 carrier and non-carrier NSDE (ocular phenotype). Note: *P < 0.05; **P < 0.01; ****P < 0.0001; ns, no significance.

Next, we determined whether the expression of HLA susceptibility alleles was associated with different clinical manifestations in the NSDE group and the SSDE group. In the NSDE group, no significant difference was detected for age or sex between the HLA-B*40:01 carrier and the non-carrier group (Table 4). There was a trend toward worsening the ocular surface condition with the strongest trend being for B*40:01. This was manifested by a higher OSDI score (53.58 vs. 50.94), worse VA (0.15 vs. 0.00), lower Schirmer test score (3.5 vs. 4.0), lower TMH (0.12 vs. 0.13), shorter NIBUT (3.44 vs. 4.50), higher bulbar redness score (1.15 vs. 1.10), greater MGDS (0.80 vs. 0.57), and higher CFS score (4 vs. 2), and there were statistically significant differences in the VA, MGDS, and CFS scores (Fig. 1). In addition, patients were further divided into moderate and severe, according to the outcome of Oxford CFS scoring scheme with a cutoff > level 2. Further analysis of the data revealed that the higher severe rate was associated with HLA-B*40:01 carrier status (67.39 vs. 42.68), as shown in Figure 1i. Figures 1j, and 1k show typical slit lamp photographs after fluorescein dye instillation of HLA-B*40:01 carrier and non-carrier patients.

**Table 4.** Comparison of Clinical Manifestations Between HLA-B*40:01 Carriers and Non-Carriers in Patients With Non-Sjögren's Syndrome Dry Eye

	Non-Sjögren's Syndrome Dry Eye (HLA-B*40:01)		
Variables	Carrier (*N* = 23, 46 Eyes)	Non-Carrier (*N* = 41, 82 Eyes)	*P* Value
Age, mean (SD), y	50.09 (9.71)	46.12 (11.07)	0.156*
Female, *N* (%)	22 (95.7%)	37 (90.2%)	0.646^†^
OSDI score, mean (SD)	53.58 (25.14)	50.94 (20.78)	0.655*
VA, median (IQR), LogMAR	0.15 (0.00–0.22)	0.00 (0.00–0.22)	0.006^‡^
Schirmer test, median (IQR), mm/5 min	3.50 (2.25–5.00)	4.00 (2.00–7.00)	0.288^‡^
TMH, median (IQR), mm	0.12 (0.11–0.16)	0.13 (0.09–0.18)	0.880^‡^
NIBUT, median (IQR), s	3.44 (2.87–5.57)	4.50 (3.20–7.26)	0.115^‡^
Bulbar redness score, median (IQR)	1.15 (0.90–1.50)	1.10 (0.90–1.35)	0.901^‡^
MGDS, mean (SD)	0.80 (0.62)	0.57 (0.55)	0.030^‡^
CFS score, median (IQR)	4.00 (2.00–4.00)	2.00 (1.00–3.25)	<0.0001^‡^

* Independent sample *t* test.

† *χ^2^* test.

‡ Mann-Whitney *U* test.

**Figure 2.**
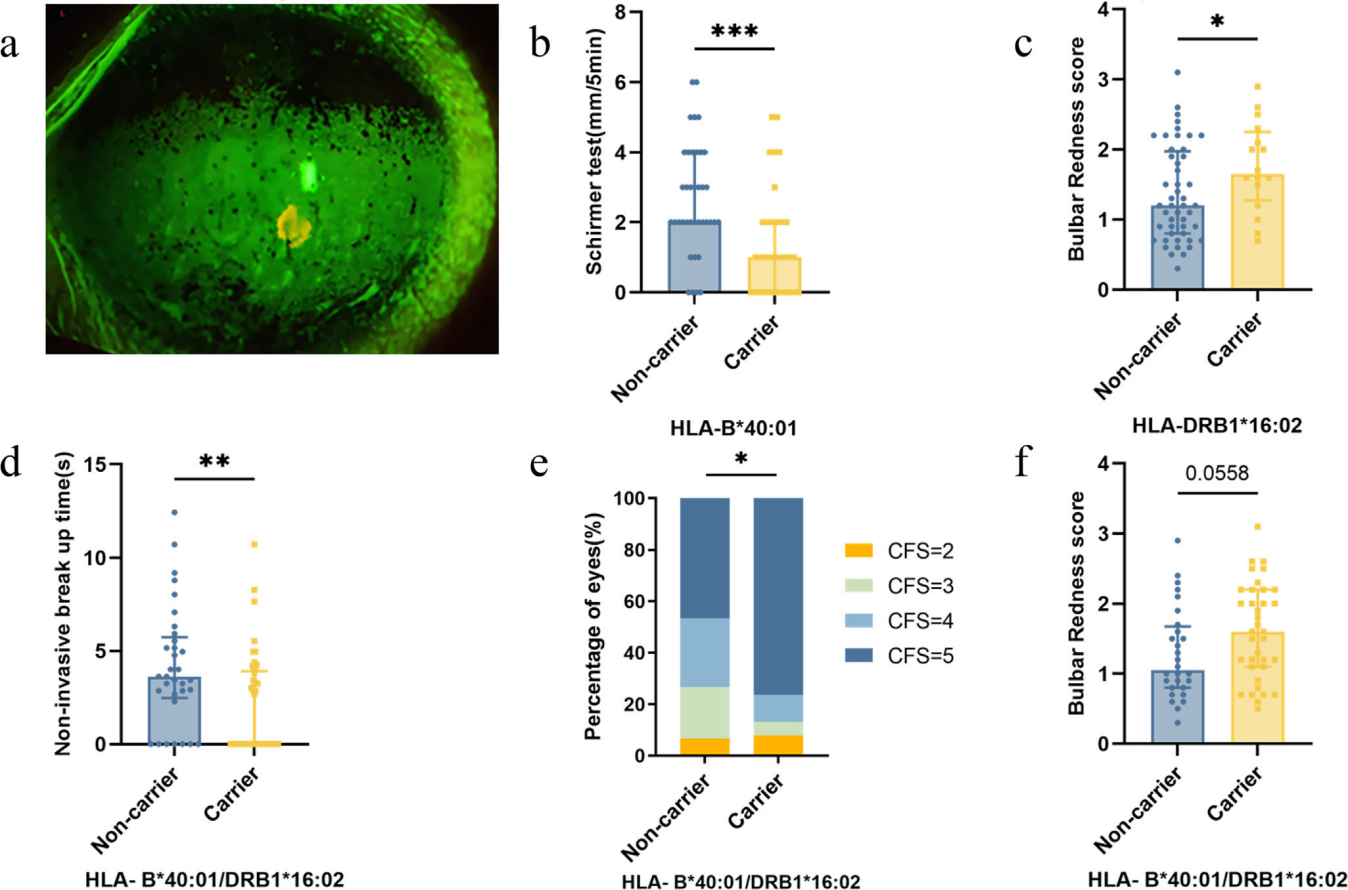
Comparison of clinical parameters in individuals with the susceptibility alleles and those without. (**a**) Representative images of fluorescein staining (Fluorescein) in patients with Sjögren's syndrome dry eye (ocular phenotype); (**b**) comparison of Schirmer test score between patients with HLA-B*40:01 carrier and non-carrier in Sjögren’s syndrome dry eye group (*P* = 0.001); (**c**) comparison of bulbar redness score between patients with HLA-DRB1*16:02 carrier and non-carrier in the Sjögren’s syndrome dry eye group (*P* = 0.040); (**d, e, f**) comparison of noninvasive break up time corneal fluorescein staining (CFS) score, bulbar redness score between patients with HLA-B*40:01 or DRB1*16:02 carrier and non-carrier in Sjögren’s syndrome dry eye group (*P* = 0.004; *P* = 0.023; and *P* = 0.056). Note: *P < 0.05; **P < 0.01; ***P < 0.001.

In the SSDE group, comparison of clinical parameters in HLA-B*40:01 carriers and non-carriers (Supplementary Table S3) revealed that a lower Schirmer test score was associated with HLA-B*40:01 (Fig. 2b). HLA-DRB1*16:02 individuals experienced more severe conjunctival congestion, with higher bulbar redness score (see Supplementary Table S3, Fig. 2c). For HLA-C*07:02 and HLA-DQB1*06:01, no statistically significant difference was detected between the carriers and the non-carriers (see Supplementary Table S3). When HLA-B*40:01 and HLA-DRB1*16:02 were considered together, we identified significantly shorter NIBUT and higher CFS scores in individuals with the susceptibility alleles compared with those without. The carrier group was also observed to exhibit a trend toward a higher bulbar redness score, but this difference was not significant (see Supplementary Table S3, Figs. 2d–f).

## Discussion

This study demonstrates a potential association of HLA polymorphisms with moderate-to-severe DED, both in individuals with and without SS. Noninfectious inflammation based on immune abnormalities is an important part of the complex pathogenesis of DED.^13^ Existing studies suggest that, in addition to SSDE, NSDE is also an autoimmune ocular surface disease,^29^ characterized by immune inflammation.^16^^,^^30^^,^^31^ The HLA system is the most important genetic susceptibility factor for immune inflammation. HLA gene variation has been linked to the greatest number of human diseases that have an immunological component.^9^ Nonetheless, very little was found in the literature about HLA in regard to DED.

The results showed that the frequency of HLA-B*40:01 was significantly higher in patients with moderate-to-severe NSDE. Multiple lines of evidence support a genetic disposition for the development of moderate-to-severe DED. An analysis of polymorphic markers revealed the association of *TRIM21* gene and *PTPN22* gene polymorphisms with the risk of developing exogenous dry eye.^32^ Ren et al. showed that the frequency of the combination of KIR2DS2 and the HLA-C1 allele group was significantly higher in patients with severe DED compared with the controls.^33^ According to the result of this study, NSDE individuals expressing HLA-B*40:01 had a higher proportion of severe and poorer ocular surface condition, as evidenced by higher CFS score, more severe meibomian gland dropout, and worse VA. The result of this study suggests that HLA-B*40:01 might be a susceptibility allele for moderate-to-severe NSDE, indicating that HLA is involved in the pathogenesis of moderate-to-severe NSDE.

This study determined that the susceptibility genes in SSDE include HLA-B*40:01, HLA-C*07:02, HLA-DQB1*06:01, and HLA-DRB1*16:02. In fact, previous studies have identified some specific HLA polymorphisms that are linked to SS. The study by Kang et al. reported different HLA alleles associated with SS in different ethnic groups, with susceptibility genes in Chinese patients with SS, including HLA-DRB1*08:03, HLA-DQA1*01:03, and HLA-DQB1*06:01, and consistent with our findings.^34^ A GWAS has identified strong associations with SS in the HLA region. Among them, HLA-DR and HLA-DQ were the key genetic determinants of SS susceptibility, with the peak effect.^35^ It is noteworthy that our study is the first to report the associations of HLA-DRB1*16:02, HLA-B*40:01, and C*07:02 and with SSDE. Notably, our study identified HLA-DRB1*16:02 as a promising marker, with a high odds ratio of 5.06. What’s more, our study revealed that SSDE individuals who possessed the HLA-DRB1*16:02 allele displayed more pronounced conjunctival congestion. Extensive research has shown that HLA-DRB1*16:02 is linked to a range of autoimmune disorders, including systemic lupus erythematosus, Graves’ disease, and neuromyelitis optica.^36^^,^^37^ Based on previous studies and this study, multiallelic HLA associations may also be important in SSDE, and our study specifically identifies HLA-DRB1*16:02 as a promising marker for SSDE.

This research aimed to seek similarities between NSDE and SSDE. Among the susceptibility genes in SSDE, HLA-B*40:01 is a common locus for NSDE, suggesting a shared genetic background. In addition, clinical severity differed between carriers and non-carriers in both groups: in the NSDE group, differences were observed in VA, MGDS, and CFS scores; while in the SSDE group, the Schirmer test score showed a difference. In reviewing the literature, HLA-B*40:01 has been implicated in several other autoimmune and inflammatory diseases. Specifically, HLA-B*40:01 has been identified as a risk factor for ankylosing spondylitis, alongside HLA-B*27 alleles.^38^^,^^39^ HLA-B*40:01 indicated an inferior result from immunosuppressive therapy in patients with aplastic anemia (AA) and correlated with late-onset of AA.^40^^,^^41^ HLA-B*40:01 has also been shown to contribute to the risk of phenytoin-induced cutaneous adverse drug reactions.^42^ As outlined previously, dry eye precedes ocular and systemic complications on average by 1 decade for patients with SS. Of affected patients, 16.7% had not been diagnosed when they presented to an ophthalmologist and were diagnosed during follow-up. Fifty percent of the patients were diagnosed at the initial evaluation. Our finding suggests the possibility of a progressive relationship between NSDE and SSDE. Delayed diagnosis and treatment of SS increases the risk of systemic involvement. We strongly recommend follow-up for Sjögren-specific autoantibodies in patients with moderate-to-severe NSDE. In our future research, we plan to prospectively follow-up patients with moderate-to-severe NSDE to observe whether they will progress to SSDE.

In this study, healthy non-DED individuals were not recruited as a control group, because some of the healthy non-DED individuals may subsequently have DED beyond this period. Therefore, control data were derived from a large sample representative database. Due to the lack of dry eye clinical characteristics, an extremely small number of our control group may have moderate-to-severe DED. If so, the HLA associations described would be an underestimate. HLA associations with moderate-to-severe DED may therefore be stronger than we described.^43^

As with all studies, when interpreting the results, study limitations should be considered. First, our study population consisted of a Chinese Han population. Differences in demographic factors, such as racial distribution, may affect the generalizability of our results to other populations. Large-scale studies involving diverse ethnic populations are required to further validate these associations. Second, HLA gene polymorphisms result in functional differences in expressed HLA molecules, that is presentation of peptide antigens to T lymphocytes, and showed interindividual differences in immune responses to antigens.^44^ Our study did not explore the molecular mechanisms and future functional studies are warranted. Subgroup analysis based on serological characteristics and immunological parameters may be helpful in teasing out allele and severity of clinical manifestation correlation.

In conclusion, our study identifies HLA-B*40:01 as a common susceptibility allele for both non-Sjögren's and Sjögren's syndrome dry eye (NSDE and SSDE, respectively). Additionally, HLA-DRB1*16:02 is identified as a specific susceptibility allele for SSDE. Our study contributes to the understanding of the genetic underpinnings of these conditions and may have implications for genetic polymorphism-based clinical diagnosis and targeted therapeutic interventions for DED.

## Supplementary Material

Supplement 1
