# Supplementary material for: Association of Human Leukocyte Antigen Polymorphisms With Moderate-to-Severe Dry Eye With and Without Sjögren's Syndrome
Source: Invest Ophthalmol Vis Sci. 2025 Jul 22;66(9):57. doi: 10.1167/iovs.66.9.57 (PMC12302039; doi:10.1167/iovs.66.9.57)
Supplement: Supplement 1 [file iovs-66-9-57_s001.pdf]

eTable1 Complete List of All Alleles at the HLA-A, HLA-B, HLA-C, HLA-DQA1, HLA-DQB1, HLA-DPA1, HLA-DPB1, HLA-DRB1, and HLA-DRB3-5 Full Loci of the Non-Sjögren's Syndrome Dry Eye Group.

Allele	Frequency N (%)	Allele	Frequency, N (%)	Allele	Frequency, N (%)	Allele	Frequency, N (%)	Allele	Frequency, N (%)
HLA-A		B*38:15	1 (0.78%)	C*15:02:01	5 (3.91%)	DPA1*02:02:02	62 (48.44%)	DRB1*12:10	1 (0.78%)
A*01:01:01	3 (2.34%)	B*39:01:01	3 (2.34%)	C*15:05:02	1 (0.78%)	DPA1*02:07:01	6 (4.69%)	DRB1*13:02:01	5 (4.24%)
A*02:01:01	10 (7.81%)	B*40:01:02	26 (20.31%)	HLA-DQA1		DPA1*02:10	1 (0.78%)	DRB1*13:12:01	1 (0.78%)
A*02:03:01	7 (5.47%)	B*40:02:01	1 (0.78%)	DQA1*01:01:01	6 (4.69%)	DPA1*04:01:01	2 (1.56%)	DRB1*14:54:01	3 (2.34%)
A*02:05:01	1 (0.78%)	B*40:06:01	2 (1.56%)	DQA1*01:01:08	1 (0.78%)	HLA-DPB1		DRB1*15:01:01	15 (11.72%)
A*02:06:01	5 (3.91%)	B*40:43	1 (0.78%)	DQA1*01:02:01	18 (14.06%)	DPB1*02:01:02	22 (18.64%)	DRB1*15:02:01	3 (2.34%)
A*02:07:01	9 (7.03%)	B*44:02:01	2 (1.56%)	DQA1*01:02:02	9 (7.03%)	DPB1*02:02:01	10 (8.47%)	DRB1*16:02:01	5 (4.24%)
A*03:01:01	5 (3.91%)	B*44:03:01	1 (0.78%)	DQA1*01:03:01	12 (9.38%)	DPB1*03:01:01	3 (2.54%)	HLA-DRB3-5	
A*11:01:01	39 (30.47%)	B*46:01:01	11 (8.59%)	DQA1*01:04:01	3 (2.34%)	DPB1*04:01:01	13 (11.02%)	DRB3*01:01:02	7 (5.74%)
A*11:02:01	4 (3.13%)	B*48:01:01	1 (0.78%)	DQA1*01:05:01	1 (0.78%)	DPB1*04:02:01	5 (4.24%)	DRB3*02:02:01	26 (21.31%)
A*24:02:01	21 (16.41%)	B*49:01:01	1 (0.78%)	DQA1*02:01:01	13 (10.16%)	DPB1*05:01:01	41 (34.75%)	DRB3*02:02:22	2 (1.64%)
A*24:02:17	1 (0.78%)	B*51:01:01	4 (3.13%)	DQA1*03:01:01	8 (6.25%)	DPB1*09:01:01	1 (0.85%)	DRB3*03:01:01	6 (4.92%)
A*24:02:31	1 (0.78%)	B*51:02:01	2 (1.56%)	DQA1*03:02:01	14 (10.94%)	DPB1*10:01:01	1 (0.85%)	DRB3*03:01:03	17 (13.93%)
A*26:01:01	3 (2.34%)	B*52:01:01	1 (0.78%)	DQA1*03:03:01	5 (3.91%)	DPB1*13:01:01	3 (2.54%)	DRB4*01:03:01	33 (27.05%)
A*29:01:01	1 (0.78%)	B*54:01:01	4 (3.13%)	DQA1*04:01:01	1 (0.78%)	DPB1*14:01:01	4 (3.39%)	DRB4*01:03:02	8 (6.56%)
A*30:01:01	2 (1.56%)	B*57:01:01	1 (0.78%)	DQA1*05:01:01	9 (7.03%)	DPB1*17:01:01	4 (3.39%)	DRB5*01:01:01	20 (16.39%)
A*31:01:02	3 (2.34%)	B*58:01:01	11 (8.59%)	DQA1*05:03:01	1 (0.78%)	DPB1*19:01:01	3 (2.54%)	DRB5*01:02:01	1 (0.82%)
A*32:01:01	2 (1.56%)	B*81:02:01	1 (0.78%)	DQA1*05:05:01	10 (7.81%)	DPB1*21:01	3 (2.54%)	DRB5*01:03	1 (0.82%)
A*33:03:01	11 (8.59%)	HLA-C		DQA1*05:06:01	2 (1.56%)	DPB1*36:01	1 (0.85%)	DRB5*01:08:01N	1 (0.82%)
HLA-B		C*01:02:01	17 (13.28%)	DQA1*05:08	1 (0.78%)	DPB1*38:01	1 (0.85%)		
B*07:02:01	3 (2.34%)	C*02:02:02	2 (1.56%)	DQA1*06:01:01	14 (10.94%)	DPB1*41:01:01	1 (0.85%)		
B*07:05:01	1 (0.78%)	C*03:02:02	10 (7.81%)	HLA-DQB1		DPB1*107:01	2 (1.69%)		
B*08:01:01	1 (0.78%)	C*03:03:01	6 (4.69%)	DQB1*02:01:01	9 (7.03%)	HLA-DRB1			
B*13:01:01	7 (5.47%)	C*03:04:01	14 (10.94%)	DQB1*02:02:01	7 (5.47%)	DRB1*01:01:01	5 (3.91%)		
B*13:02:01	5 (3.91%)	C*04:01:01	7 (5.47%)	DQB1*03:01:01	32 (25.00%)	DRB1*03:01:01	9 (7.03%)		
B*15:01:01	7 (5.47%)	C*04:03:01	4 (3.13%)	DQB1*03:02:01	8 (6.25%)	DRB1*04:03:01	1 (0.78%)		
B*15:02:01	10 (7.81%)	C*05:01:01	1 (0.78%)	DQB1*03:03:02	15 (11.72%)	DRB1*04:04:01	1 (0.78%)		
B*15:05:01	1 (0.78%)	C*06:02:01	6 (4.69%)	DQB1*04:01:01	4 (3.13%)	DRB1*04:05:01	4 (3.13%)		
B*15:11:01	2 (1.56%)	C*07:01:01	2 (1.56%)	DQB1*04:02:01	2 (1.56%)	DRB1*04:06:01	6 (4.69%)		
B*15:13:01	1 (0.78%)	C*07:02:01	28 (21.88%)	DQB1*05:01:01	6 (4.69%)	DRB1*04:10:01	1 (0.78%)		
B*15:25:01	2 (1.56%)	C*07:04:01	2 (1.56%)	DQB1*05:01:24	1 (0.78%)	DRB1*07:01:01	10 (7.81%)		
B*15:27:01	2 (1.56%)	C*07:18:01	1 (0.78%)	DQB1*05:02:01	16 (12.50%)	DRB1*08:03:02	11 (8.59%)		
B*18:01:01	1 (0.78%)	C*07:56:02	1 (0.78%)	DQB1*06:01:01	17 (13.28%)	DRB1*08:09:01	1 (0.78%)		
B*27:05:02	1 (0.78%)	C*07:66	1 (0.78%)	DQB1*06:02:01	6 (4.69%)	DRB1*09:01:02	14 (10.94%)		
B*35:01:01	3 (2.34%)	C*08:01:01	14 (10.94%)	DQB1*06:04:01	1 (0.78%)	DRB1*10:01:01	1 (0.78%)		
B*35:05:01	1 (0.78%)	C*08:22:01	1 (0.78%)	DQB1*06:09:01	4 (3.13%)	DRB1*11:01:01	7 (5.47%)		
B*35:08:01	1 (0.78%)	C*12:02:02	2 (1.56%)	HLA-DPA1		DRB1*11:04:01	1 (0.78%)		
B*37:01:01	1 (0.78%)	C*14:02:01	2 (1.56%)	DPA1*01:03:01	46 (35.94%)	DRB1*12:01:01	6 (4.69%)		
B*38:02:01	4 (3.13%)	C*14:03:01	1 (0.78%)	DPA1*02:01:01	11 (8.59%)	DRB1*12:02:01	17 (13.28%)		

eTable2 Complete List of All Alleles at the HLA-A, HLA-B, HLA-C, HLA-DQA1, HLA-DQB1, HLA-DPA1, HLA-DPB1, HLA-DRB1, and HLA-DRB3-5 Full Loci of the Sjögren's Syndrome Dry Eye Group.

Allele	Frequency N (%)	Allele	Frequency N (%)	Allele	Frequency N (%)	Allele	Frequency N (%)
HLA-A		B*54:01:01	3 (3.75%)	HLA-DPA1		HLA-DRB1	
A*01:01:01	1 (1.25%)	B*55:02:01	3 (3.75%)	DPA1*01:03:01	13(16.25%)	DRB1*01:01:01	1 (1.25%)
A*02:01:01	6 (7.50%)	B*55:02:03	1 (1.25%)	DPA1*02:01:01	5 (6.25%)	DRB1*03:01:01	3 (3.75%)
A*02:03:01	5 (6.25%)	B*57:01:01	1 (1.25%)	DPA1*02:02:02	54 (67.50%)	DRB1*04:05:01	7 (8.75%)
A*02:06:01	3 (3.75%)	B*58:01:01	3 (3.75%)	DPA1*02:02:08	1 (1.25%)	DRB1*07:01:01	4 (5.00%)
A*02:07:01	7 (8.75%)	B*67:01:01	1 (1.25%)	DPA1*02:07:01	4 (5.00%)	DRB1*08:03:02	13 (16.25%)
A*11:01:01	22 (27.50%)	HLA-C		DPA1*04:01:01	3 (3.75%)	DRB1*09:01:02	14 (17.50%)
A*11:02:01	3 (3.75%)	C*01:02:01	14 (17.50%)	HLA-DQB1		DRB1*11:01:01	1 (1.25%)
A*23:01:01	1 (1.25%)	C*02:02:02	2 (2.50%)	DQB1*02:01:01	3 (3.75%)	DRB1*12:01:01	3 (3.75%)
A*24:02:01	16 (20.00%)	C*03:02:02	3 (3.75%)	DQB1*02:02:01	4 (5.00%)	DRB1*12:02:01	3 (3.75%)
A*24:02:31	1 (1.25%)	C*03:03:01	2 (2.50%)	DQB1*03:01:01	9 (11.25%)	DRB1*13:01:01	2 (2.50%)
A*24:03:01	1 (1.25%)	C*03:04:01	9 (11.25%)	DQB1*03:02:01	1 (1.25%)	DRB1*13:12:01	2 (2.50%)
A*24:07:01	1 (1.25%)	C*04:01:01	3 (3.75%)	DQB1*03:03:02	13(16.25%)	DRB1*14:18	1 (1.25%)
A*24:08	1 (1.25%)	C*04:03:01	1 (1.25%)	DQB1*04:01:01	6 (7.50%)	DRB1*15:01:01	12 (15.00%)
A*26:01:01	4 (5.00%)	C*06:02:01	5 (6.25%)	DQB1*05:01:01	1 (1.25%)	DRB1*15:02:01	3 (3.75%)
A*30:01:01	4 (5.00%)	C*07:01:01	1 (1.25%)	DQB1*05:01:24	1 (1.25%)	DRB1*16:02:01	11 (13.75%)
A*32:01:01	1 (1.25%)	C*07:02:01	27 (33.75%)	DQB1*05:02:01	14 (17.50%)	HLA-DRB3-5	
A*33:03:01	3 (3.75%)	C*07:06:01	1 (1.25%)	DQB1*05:03:01	1 (1.25%)	DRB3*01:01:02	3 (3.85%)
HLA-B		C*08:01:01	3 (3.75%)	DQB1*06:01:01	20	DRB3*02:02:01	10 (12.82%)
B*07:02:01	1 (1.25%)	C*08:03:01	1 (1.25%)	DQB1*06:02:01	5 (6.25%)	DRB3*02:02:22	2 (2.56%)
B*13:01:01	5 (6.25%)	C*12:02:02	3 (3.75%)	DQB1*06:03:01	2 (2.50%)	DRB3*03:01:03	5 (6.41%)
B*13:02:01	4 (5.00%)	C*12:03:01	2 (2.50%)	HLA-DPA1		DRB4*01:03:01	19 (24.36%)
B*15:01:01	5 (6.25%)	C*14:02:01	2 (2.50%)	DPA1*01:03:01	13(16.25%)	DRB4*01:03:02	9 (11.54%)
B*15:02:01	1 (1.25%)	C*15:02:01	1 (1.25%)	DPA1*02:01:01	5 (6.25%)	DRB5*01:01:01	23 (29.49%)
B*15:27:01	1 (1.25%)	HLA-DQA1		DPA1*02:02:02	54 (67.50%)	DRB5*01:08:01N	1 (1.28%)
B*27:04:01	2 (2.50%)	DQA1*01:01:01	2 (2.50%)	DPA1*02:02:08	1 (1.25%)	DRB5*02:02:01	4 (5.13%)
B*27:05:02	2 (2.50%)	DQA1*01:02:01	13 (16.25%)	DPA1*02:07:01	4 (5.00%)	DRB5*02:03	2 (2.56%)
B*35:05:01	1 (1.25%)	DQA1*01:02:02	11 (13.75%)	DPA1*04:01:01	3 (3.75%)		
B*38:02:01	4 (5.00%)	DQA1*01:03:01	18 (22.50%)	HLA-DPB1			
B*39:01:01	4 (5.00%)	DQA1*01:03:04	1 (1.25%)	DPB1*02:01:02	11 (16.18%)		
B*39:05:01	1 (1.25%)	DQA1*01:04:01	1 (1.25%)	DPB1*02:02:01	5 (7.35%)		
B*40:01:02	22 (27.50%)	DQA1*02:01:01	4 (5.00%)	DPB1*03:01:01	3 (4.41%)		
B*40:06:01	1 (1.25%)	DQA1*03:02:01	12 (15.00%)	DPB1*05:01:01	37 (54.41%)		
B*41:01:01	1 (1.25%)	DQA1*03:03:01	7 (8.75%)	DPB1*13:01:01	1 (1.47%)		
B*44:03:02	1 (1.25%)	DQA1*05:01:01	3 (3.75%)	DPB1*135:01	3 (4.41%)		
B*46:01:01	7 (8.75%)	DQA1*05:03:01	2 (2.50%)	DPB1*17:01:01	2 (2.94%)		
B*48:01:01	1 (1.25%)	DQA1*05:05:01	3 (3.75%)	DPB1*19:01:01	3 (4.41%)		
B*51:01:01	3 (3.75%)	DQA1*05:06:01	1 (1.25%)	DPB1*107:01	2 (2.94%)		
B*52:01:01	1 (1.25%)	DQA1*06:01:01	2 (2.50%)	DPB1*1273:01	1 (1.47%)		

eTable3 Associations Between Risk Alleles and Clinical Parameters in Sjögren's Syndrome Dry Eye Patients

	Age, mean (SD), y	Female, N (%)	OSDI score, mean (SD)	VA, median (IQR), LogMAR	Schirmer test, median(IQR), mm/5min	TMH, median (IQR), mm	NIBUT, median (IQR), s	Bulbar Redness, median (IQR)	MGDS, median (IQR)	CFS score, median (IQR)
**HLA-B*40:01**										
Carrier (N=18, 36eyes)	50.06(13.46)	15(83.3%)	61.27 (24.42)	0.22(0.05,0.28)	1.00(0.00,2.00)	0.11(0.07,0.15)	3.22(0.00,4.92)	1.20(0.73,1.98)	1.00(0.00,1.25)	5.00(4.00,5.00)
Non-carrier(N=22,44eyes)	54.82(11.56)	21(95.5%)	73.03 (16.13)	0.26(0.09,0.52)	2.00(2.00,4.00)	0.10(0.08,0.12)	2.90(0.00,4.07)	1.50(0.93,2.20)	1.00(1.00,2.00)	5.00(3.00,5.00)
P value	0.236[a]	0.310[b]	0.083[a]	0.185[c]	0.001[c]	0.412[c]	0.580[c]	0.165[c]	0.108[c]	0.549[c]
**HLA-DRB1*16:02**										
Carrier (N=10, 20eyes)	53.90 (9.74)	8 (80.0%)	71.13 (17.24)	0.22 (0.00,0.38)	1.50 (0.00,3.00)	0.10 (0.08,0.12)	2.97 (0.00,4.83)	1.65 (1.28,2.25)	1.50 (1.00,2.00)	5.00 (4.75,5.00)
Non-carrier(N=30,60eyes)	52.27(13.44)	28 (93.3%)	66.87 (21.85)	0.22 (0.10,0.52)	2.00 (1.00,3.25)	0.10 (0.07,0.14)	2.97 (0.00,4.40)	1.20 (0.80,1.98)	1.00 (1.00,1.75)	5.00 (4.00,5.00)
P value	0.726[a]	0.256[b]	0.614[a]	0.373[c]	0.265[c]	0.554[c]	0.763[c]	0.040[c]	0.094[c]	0.205[c]
**HLA-C*07:02**										
Carrier (N=24, 48 eyes)	51.71(13.28)	20(83.3%)	63.09 (21.46)	0.22(0.00,0.33)	2.00(0.25,3.00)	0.10(0.08,0.15)	2.96(0.00,5.16)	1.35(0.88,2.00)	1.00(1.00,2.00)	5.00(4.00,5.00)
Non-carrier(N=16,32eyes)	54.13(11.55)	16(100.0%)	74.94 (18.19)	0.22(0.11,0.52)	2.00(1.00,3.25)	0.10(0.08,0.12)	3.06(0.00,3.87)	1.40(0.95,2.20)	1.00(1.00,2.00)	5.00(4.00,5.00)
P value	0.557[a]	0.136[b]	0.086[a]	0.134[c]	0.183[c]	0.578[c]	0.480[c]	0.443[c]	0.959[c]	0.617[c]
**HLA-DQB1*06:01**										
Carrier (N=17, 34eyes)	50.06(14.69)	14 (82.4%)	61.04 (21.25)	0.22 (0.05,0.33)	2.00 (1.00,3.25)	0.10 (0.08,0.13)	3.35 (0.00,5.16)	1.20 (0.93,1.75)	1.00 (0.00,1.75)	5.00 (3.25,5.00)
Non-carrier(N=23,46eyes)	54.61(10.57)	22 (95.7%)	72.67 (19.53)	0.22 (0.06,0.70)	2.00 (0.25,3.00)	0.10 (0.08,0.14)	2.87 (0.00,4.01)	1.55 (0.80,2.20)	1.00 (1.00,2.00)	5.00 (4.00,5.00)
P value	0.261[a]	0.294[b]	0.089[a]	0.117[c]	0.710[c]	0.548[c]	0.223[c]	0.316[c]	0.092[c]	0.271[c]
**HLA-B*40:01/DRB1*16:02**										
Carrier (N=23,46eyes)	51.78(13.02)	19 (82.6%)	64.25 (22.99)	0.22 (0.04,0.30)	2.00 (0.00,3.50)	0.10 (0.08,0.14)	0.00 (0.00,3.92)	1.61 (0.68)	1.00 (1.00,2.00)	5.00 (4.75,5.00)
Non-carrier(N=17,34eyes)	53.88(12.09)	17 (100.0%)	72.11 (17.49)	0.31 (0.10,0.52)	2.00 (0.00,3.50)	0.10 (0.08,0.14)	3.63 (2.49,5.74)	1.28 (0.65)	1.00 (1.00,2.00)	4.00 (3.00,5.00)
P value	0.606[a]	0.123[b]	0.253[a]	0.191[c]	0.720[c]	0.935[c]	0.004[c]	0.056[a]	0.818[c]	0.023[c]

（[a] Independent sample *t* test; [b] χ^2 test; [c] Mann-Whitney U test）OSDI: ocular surface disease index; VA: visual acuity (LogMAR=logarithm of the minimal angle of resolution); TMH: tear meniscus height; NIBUT: non-invasive break up time; MGDS: meibomian gland dropout scores; CFS: Corneal fluorescein staining (Oxford scheme); SD: standard deviation; IQR: interquartile range
